# A Space-Based Autonomous Timekeeping Method Based on Onboard Atomic Clocks and Inter-Satellite Measurements

**DOI:** 10.3390/s26092635

**Published:** 2026-04-24

**Authors:** Guangyao Chen, Shanshi Zhou, Xiaogong Hu, Chengpan Tang, Junyang Pan

**Affiliations:** 1 Shanghai Astronomical Observatory, Chinese Academy of Sciences, Shanghai 200030, China; chenguangyao@shao.ac.cn (G.C.); hxg@shao.ac.cn (X.H.); cptang@shao.ac.cn (C.T.); panjy@shao.ac.cn (J.P.); 2 University of Chinese Academy of Sciences, Beijing 100049, China

**Keywords:** BeiDou navigation satellite system (BDS-3), hydrogen maser, inter-satellite link (ISL), space-based timekeeping, space-based atomic timescale

## Abstract

**Highlights:**

**What are the main findings?**

- To improve the quality of inter-satellite time transfer products for space-based timekeeping, we combine ISL network adjustment with a high-precision relativistic correction. This strategy suppresses random noise from ~0.17 ns to ~0.04 ns (≈72.1% reduction) and improves ADEV at $\tau \approx 10^{4}$ s from $2.60\times 10^{-14}$ to $2.14\times 10^{-14}$ (≈17.77% improvement).
- To evaluate constellation-level timescale maintenance under different link-availability conditions, we test both free-running TA(SPACE) and periodically steered TA(SPACE). In the free-running mode, TA(SPACE) stays within 80 ns relative to BDT over 90 days (RMS 19.69±14.24 ns; End 25.06±41.47 ns), while periodic BDT steering with 10–30 d updates maintains the TA(SPACE)–BDT offset within ~10 ns.

**What are the implications of the main findings?**

- A constellation equipped with onboard hydrogen masers and precise ISL time transfer can establish and maintain a self-consistent space-based timescale, enabling a practical pathway from space–ground cooperative timekeeping toward constellation-level autonomy.
- The proposed architecture and methods provide a scalable framework for resilient timing services, supporting robust time synchronization and clock prediction even when ground contact is limited.

**Abstract:**

In global navigation satellite systems (GNSS), the system time reference is maintained by the ground control segment and kept traceable to UTC, enabling inter-system compatibility and interoperability. Advances in onboard atomic-clock stability and inter-satellite time transfer accuracy make it feasible for a constellation to autonomously realize a space-based time reference, with periodic traceability updates and steering via satellite–ground links to enhance resilient time maintenance. BeiDou-3 (BDS-3) carries high-performance onboard hydrogen masers and Ka-band inter-satellite links (ISL) for time transfer, providing stable frequency sources and high-precision time transfer capability for establishing a space-based time reference. Using in-orbit BDS-3 clock offset data, we propose a space-based autonomous timekeeping approach that combines high-precision ISL synchronization with timekeeping by a small ensemble of hydrogen masers, together with a space–ground cooperative strategy with BeiDou time (BDT). The approach first performs constellation-wide synchronization using ISL, then selects a timekeeping ensemble based on in-orbit clock performance to generate a space-based ensemble atomic timescale, denoted TA(SPACE); when satellite–ground links are available, TA(SPACE) is steered to BDT to maintain consistency with the ground time reference. Based on this space-based time reference, satellite clock offsets are predicted to generate clock-parameter products. Experiments show that, in the autonomous mode, the time offset between TA(SPACE) and BDT is kept within 25.06 ± 41.47 ns over 90 days, whereas in the space–ground cooperative mode, satellite–ground steering stabilizes the offset within 10 ns. The proposed approach provides a practical solution for constellation time maintenance under disruptions such as anomalous ground injection, improving the resilience and reliability of GNSS services.

## 1. Introduction

The accuracy and stability of the system time reference underpin high-precision navigation, positioning, and timing (PNT) services. To ensure a stable and reliable system timescale, global navigation satellite systems (GNSS) commonly employ an ensemble clock architecture that combines spaceborne and ground-based atomic clocks. For example, GPS introduced an ensemble clock algorithm in its early development stage [1,2], in which spaceborne and ground atomic clocks are jointly used to maintain GPS Time (GPST). Ground hydrogen masers provide long-term stability and maintain traceability to Coordinated Universal Time (UTC), whereas onboard atomic clocks provide short-term stability and act as redundant backups, thereby improving the availability and resilience of the system timescale. Similarly, Galileo System Time (GST) is generated by combining spaceborne and ground-based atomic clocks at the Precise Timing Facility (PTF) and is constrained in the long term through comparisons with UTC [3]. More recently, robust timing architectures for next-generation Galileo System Time generation have also been investigated, with particular emphasis on fault tolerance, continuity, and resilient ensemble clock operation [4,5]. Studies on future integrated PNT architectures spanning high-, medium-, and low-altitude orbits likewise regard ensemble clocks as a fundamental approach for system time maintenance [6].

With advances in space atomic clocks, establishing and maintaining a space-based timescale is becoming a key capability for space missions. NASA’s Deep Space Atomic Clock (DSAC) demonstrated a ~3 × 10^−15^ frequency stability over 23 days without drift removal, with a drift rate of 3.0(0.7) × 10^−16^ per day, outperforming current in-orbit atomic clocks [7]. In ground tests, a cold-atom microwave clock achieved a long-term frequency stability of 2.5 × 10^−15^ at τ = 2 × 10^5^ s. The clock is planned to be deployed on the Chinese space station’s Mengtian laboratory module, where an in-orbit stability of approximately (7.1–9.4) × 10^−14^/√τ is expected [8]. These results indicate that the microgravity and low-noise environment in near-Earth space provides favorable conditions for atomic clocks. However, in-orbit demonstrations still focus mainly on single-clock timekeeping, and systematic studies on constellation-level timescale generation and long-term maintenance using multiple spaceborne clocks remain relatively limited, although several recent studies have begun to explore autonomous and distributed space-based timekeeping architectures [9,10,11].

BeiDou-3 (BDS-3) provides an important in-orbit platform for research on a space-based timescale. BDS-3 satellites are equipped with high-performance onboard atomic clocks. The onboard hydrogen masers achieve frequency stability on the order of 10^−15^ at τ = 1 day, while the onboard rubidium clocks reach the 10^−14^ at τ = 10,000 s [12,13]. BDS-3 also supports Ka-band inter-satellite measurement links, enabling sub-nanosecond inter-satellite time transfer. Reported assessments indicate a measurement uncertainty of 0.2 ns or better [14,15,16]. Together, highly stable onboard clocks and high-precision inter-satellite time transfer provide the foundation for constructing a space-based timescale.

At present, BDS-3 adopts an architecture in which precise orbit determination and time synchronization are processed independently. BeiDou time (BDT) is mainly maintained by hydrogen masers at the ground control segment, and satellite clock offsets are measured and estimated using the ground master clock [17]. This architecture provides direct measurements referenced to the ground-maintained BDT. However, affected by satellite–ground time transfer links, tracking-network geometry, and master-clock operation, the estimated onboard clock offsets still exhibit larger random noise and systematic errors than the level achievable with inter-satellite time transfer measurements. Therefore, the current architecture does not fully exploit onboard hydrogen masers and inter-satellite links to build a time reference that is primarily space-based and constrained by the ground. Under the current BDS-3 architecture, a key question is how to construct a space-based timescale using onboard atomic clocks and inter-satellite links. Another challenge is to maintain this timescale over long durations. It is also necessary to integrate it with BDT to form a complementary space–ground cooperative timekeeping architecture.

To address these needs, we build on recent progress in the literature and the current in-orbit status of BDS-3. We propose a space-based timekeeping approach based on onboard hydrogen masers and inter-satellite links, and present a cooperative operating strategy integrated with BDT. The remainder of this paper is organized as follows. Section 2 describes the proposed space-based timekeeping method. Section 3 presents the performance evaluation of the BDS-3 onboard atomic clocks. Section 4 analyzes the accuracy of the space-based timescale and the clock offset prediction performance using in-orbit measurements and evaluates the resulting improvements in navigation services. Section 5 concludes the paper and outlines future work.

## 2. Space-Based Timekeeping Method

### 2.1. Satellite–Ground and Inter-Satellite Time Transfer Method

The BDS-3 Ka-band ISL uses a dual one-way time-division multiple access (TDMA) measurement mode. Within one polling cycle, two mutually visible satellites (A and B) perform forward and backward one-way measurements in sequence. A linear combination of the two one-way observables is used to form a two-way time transfer equation. The basic observation model in a single time slot can be written as [18,19]:

(1) $$\rho_{AB}(t_{1})=|r_{B}(t_{1})-r_{A}(t_{1}-∆t_{1})|+c({clk}_{B}(t_{1})-{clk}_{A}(t_{1}-∆t_{1})+\tau_{A}^{send}+\tau_{B}^{rcv})+\delta_{AB}+\varepsilon_{AB}$$

(2) $$\rho_{BA}(t_{2})=|r_{A}(t_{2})-r_{B}(t_{2}-∆t_{2})|+c({clk}_{A}(t_{2})-{clk}_{B}(t_{2}-∆t_{2})+\tau_{A}^{rcv}+\tau_{B}^{send})+\delta_{BA}+\varepsilon_{BA}$$

Here, ρ denotes the one-way pseudorange observation. $t_{1}$ and $t_{2}$ are the signal transmission epochs, and r is the satellite position vector. $\Delta t_{1}$ and $\Delta t_{2}$ denote the corresponding signal propagation times, and c is the speed of light. ${clk}_{A}$ and ${clk}_{B}$ are the satellite clock offsets. $\tau^{send}$ and $\tau^{rcv}$ represent the ISL payload transmit and receive hardware delays. $\delta_{AB}$ and $\delta_{BA}$ are the correction terms, including antenna phase center corrections and relativistic corrections. For satellite–ground links, they further include tropospheric delay, station eccentricity, and tidal corrections. ε denotes the measurement noise and other unmodeled errors.

Due to the TDMA scheme, the transmission epochs $t_{1}$ and $t_{2}$ are not identical. To form a two-way time transfer equation at a common epoch, the observations are reduced to the start of the polling cycle, $t_{0}$. Let the reduced pseudoranges be $\rho_{AB}(t_{0})$ and $\rho_{BA}(t_{0})$. Then, we have:

(3) $$\rho_{AB}\left(t_{0}\right)=\rho_{AB}\left(t_{1}\right)+d\rho_{AB}$$

(4) $$\rho_{BA}\left(t_{0}\right)=\rho_{BA}\left(t_{2}\right)+d\rho_{BA}$$

Here, $d\rho_{AB}$ and $d\rho_{BA}$ are the correction terms that reduce the observations from epochs $t_{1}$ and $t_{2}$ to $t_{0}$. They can be computed using predicted satellite orbits and clock offset models. Their accuracy mainly depends on the prediction accuracy of the satellite orbital velocity and the clock-rate (frequency) model [20]:

(5) $$\begin{array}{c} d\rho_{AB}=\left|r_{B}\left(t_{0}\right)-r_{A}\left(t_{0}\right)\right|-\left|r_{B}\left(t_{1}\right)-r_{A}\left(t_{1}-∆t_{1}\right)\right|\\ +c\left({clk}_{B}\left(t_{1}\right)-{clk}_{A}\left(t_{1}\right)\right)-c({clk}_{B}\left(t_{0}\right)-{clk}_{A}\left(t_{0}\right)) \end{array}$$

(6) $$\begin{array}{c} d\rho_{BA}=\left|r_{A}\left(t_{0}\right)-r_{B}\left(t_{0}\right)\right|-\left|r_{B}\left(t_{2}\right)-r_{A}\left(t_{2}-∆t_{2}\right)\right|\\ +c\left({clk}_{A}\left(t_{2}\right)-{clk}_{B}\left(t_{2}\right)\right)-c({clk}_{B}\left(t_{0}\right)-{clk}_{A}\left(t_{0}\right)) \end{array}$$

At the common epoch $t_{0}$, the two reduced pseudorange measurements are linearly combined. By taking their difference, the first-order geometric range term is eliminated. This yields the inter-satellite two-way time transfer observation equation:

(7) $${clk}_{A}\left(t_{0}\right)-{clk}_{B}\left(t_{0}\right)=\frac{\rho_{BA}\left(t_{0}\right)-\rho_{AB}\left(t_{0}\right)}{2c}+\frac{\delta_{AB}-\delta_{BA}}{2c}+\frac{\tau_{A}^{send}-\tau_{A}^{rcv}}{2}-\frac{\tau_{B}^{send}-\tau_{B}^{rcv}}{2}+\frac{\varepsilon_{AB}-\varepsilon_{BA}}{2}$$

Redundant ISL measurements can further suppress the random errors in inter-satellite clock offset estimation. For inter-satellite clock offsets reduced to the common epoch $t_{0}$, one satellite is selected as the reference satellite (ref). Its clock offset with respect to the system time (e.g., BDT) is treated as known or tightly constrained. The unknown parameters are the clock offsets of the remaining N−1 satellites relative to ref, collected in:

(8) $$x=\left[x_{A}^{ref},x_{B}^{ref},\ldots ,x_{N-1}^{ref}\right]$$

For an inter-satellite clock offset observation ${clk}_{i}(t_{0})-{clk}_{j}(t_{0})$, the observation equation is:

(9) $$y_{ij}={clk}_{i}^{ref}\left(t_{0}\right)-{clk}_{j}^{ref}\left(t_{0}\right)+v_{ij}$$

For a single-epoch network adjustment, the stacked form is:

(10) y=Hx+v

Here, H is the design matrix composed of $\left[1,-1,0\right]$-type patterns. Each row contains the partial derivatives for one inter-satellite observation [21]:

(11) $$\left\{\begin{array}{c} \frac{\partial ({clk}_{A}\left(t_{0}\right)-{clk}_{B}\left(t_{0}\right))}{\partial x}=[1,-1,0,\ldots ,0]\\ \ldots \\ \frac{\partial ({clk}_{A}\left(t_{0}\right)-{clk}_{ref}\left(t_{0}\right))}{\partial x}=[1,0,0,\ldots ,0]\\ \ldots \\ \frac{\partial ({clk}_{B}\left(t_{0}\right)-{clk}_{ref}\left(t_{0}\right))}{\partial x}=[0,1,0,\ldots ,0]\\ \ldots \end{array}\right.$$

With the reference satellite constraint, x is estimated by least squares. The network adjustment exploits redundant ISL observations to strengthen the network constraints and average down noise. It reduces the impact of single-link random noise and yields a high-precision clock offset series. Published assessments show that ISL can reduce the residual RMS of onboard clock offset products to about 0.16 ns, outperforming external clock products derived from global ground networks [22].

On this basis, satellite–ground time transfer links are introduced to obtain the clock offset information of visible satellites with respect to the ground reference time. Satellite–ground time transfer typically has a nanosecond-level noise. It is also more sensitive to hardware biases and propagation residuals. If a full-constellation ground-synchronization strategy is applied directly, the extra noise from the satellite–ground links will be injected into the onboard clock products and may offset the advantage of inter-satellite links. Therefore, we use the following strategy. During initialization, satellite–ground time transfer results are used to set the initial phase and frequency reference of the space-based timescale with respect to BDT. In long-term analysis, the satellite–ground results are treated as external truth to evaluate the long-term bias and drift of space-based autonomous timekeeping. This strategy effectively suppresses the impact of satellite–ground noise on the construction and evaluation of the space-based timescale.

### 2.2. Relativistic Correction Method

In high-precision timescale construction, relativistic effects induced by gravitational potential and orbital motion cannot be neglected. The International Earth Rotation and Reference Systems Service (IERS) provides the relationship between proper time and coordinate time. The formulation remains valid at the 10^−18^ accuracy level [23]:

(12) $$\frac{d\tau_{A}}{dTT}=1+L_{G}-\frac{1}{c^{2}}\left[\frac{v_{A}^{2}}{2}+U_{E}\left(x_{A}\right)+V\left(X_{A}\right)-V\left(X_{E}\right)-x_{A}^{i}\partial_{i}V\left(X_{E}\right)\right]$$

Here, $\tau_{A}$ is the proper time of the satellite located at position $x_{A}$, and TT denotes terrestrial time. $L_{G}$ is the defining constant relating TT to geocentric coordinate time (TCG). $v_{A}$ is the coordinate velocity. $U_{E}(x_{A})$ is the Newtonian gravitational potential of the Earth in the geocentric frame. V denotes the sum of Newtonian potentials of other bodies (mainly the Sun and the Moon), evaluated using the Earth’s barycentric position $X_{E}$ and the satellite barycentric position $X_{A}$. The term $x_{A}^{i}\partial_{i}V(X_{E})$ represents the potential gradient contribution associated with the transformation between the geocentric frame and the inertial frame.

Current GNSS implementations use the standard (ICD) relativistic correction. It approximately compensates the velocity term and the geocentric gravitational potential term. This approximation performs well at the $10^{-15}$ stability level. As measurement precision improves, residual higher-order terms—such as the Earth’s nonspherical potential ($J_{2}$) and the lunisolar tidal potential—accumulate over daily timescales and can reach the sub-nanosecond level [24]. These residuals reduce the modelability of the clock offset series and amplify extrapolation errors [25]. Therefore, we apply a high-precision relativistic correction to the inter-satellite clock offset series. The correction is computed as follows:

(13) $${∆t}_{r}=-\frac{1}{c^{2}}(\frac{v_{A}^{2}}{2}+\frac{GM_{E}}{r_{A}}+R+V_{S}+V_{M})$$

Here, $V_{S}$ and $V_{M}$ are the tidal potentials induced by the Sun and the Moon, and R denotes the Earth’s nonspherical gravitational potential. Previous studies have shown that correcting this error can improve prediction accuracy by about 10%. It is also important for future high-precision atomic clocks, such as optical clocks, and for high-accuracy time synchronization [26].

### 2.3. Construction Method for a Space-Based Timescale Based on Onboard Hydrogen Masers

A stable space-based timescale requires combining multiple onboard atomic clocks to achieve higher long-term stability and reliability than any single clock. The Bureau International des Poids et Mesures (BIPM) uses the ALGOS to generate the free atomic timescale EAL. It maintains the timescale robustly through an iterative process of prediction and weighting [27,28]. In this work, we extend the same idea to the constellation level. We use high-precision relative clock offsets obtained from inter-satellite time transfer to construct a space-based timescale, denoted TA(space), and then derive clock-parameter products.

Let ${clk}_{ij}$ denote the relativistically corrected inter-satellite relative clock offset, and let the space-based timescale be TA(space). The satellite clock offset with respect to TA(space) can then be written as:

(14) $$X_{space}=\left[\begin{array}{c} {clk}_{i}-TA(space)\\ \vdots \\ {clk}_{j}-TA(space) \end{array}\right]$$

ALGOS mainly consists of prediction and weighting. It is designed to improve the long-term stability and reliability of the timescale.

To prevent time and frequency discontinuities caused by adding or removing clocks across updates, the prediction step enforces the continuity of TA(space) between adjacent processing intervals. Assuming that the phase, frequency, and frequency drift of the participating clocks remain smooth over two consecutive intervals, the following constraints can be introduced:

(15) $$Z_{const}=\left[\begin{array}{c} \sum_{k=0}^{n}w_{k}\cdot {\left({clk}_{i}-TA\left(space\right)\right)}_{ph,pre}\\ \sum_{k=0}^{n}w_{k}\cdot {\left({clk}_{i}-TA\left(space\right)\right)}_{fr,pre}\\ \sum_{k=0}^{n}w_{k}\cdot {\left({clk}_{i}-TA\left(space\right)\right)}_{dr,pre} \end{array}\right]$$

Here, the subscript ph denotes the phase series, fr the frequency series, and dr the frequency-drift series. The superscript pre denotes the predicted value, and $w_{k}$ is the weight.

The weight update emphasizes clock predictability rather than short-term noise alone. Hydrogen masers often exhibit noticeable drift. However, their drift uncertainty is small, and their behavior is highly predictable. As a result, they typically receive larger weights over long arcs and dominate the long-term performance of the timescale. For clock i, the frequency variations over the most recent update windows (e.g., 5–12 updates) are used as the weighting metric. The computation is:

(16) $$\left\{\begin{array}{c} \epsilon_{i,I_{k}}=\left|y_{i,I_{k}}-{\hat{y}}_{i,I_{k}}\right|\\ \sigma_{i}^{2}=\frac{\sum_{j=1}^{M}(\frac{M+1-j}{M})\epsilon_{i,j}^{2}}{\sum_{j=1}^{M}\left(\frac{M+1-j}{M}\right)}\\ w_{i,temp}=\frac{\frac{1}{\sigma_{i}^{2}}}{\sum_{i=1}^{N}\left(\frac{1}{\sigma_{i}^{2}}\right)} \end{array}\right.$$

Here, $\epsilon_{\left(i,I_{k}\right)}$ is the prediction error of clock i, M is the window length (5≤M≤12), and $w_{\left(i,temp\right)}$ is the normalized weight. In normal cases, we set $w_{i}=w_{\left(i,temp\right)}$. If $w_{\left(i,temp\right)}$ exceeds the upper bound, we cap it at $w_{max}$. If the difference between the measured and predicted frequency of clock i exceeds a threshold, the clock is temporarily excluded or down-weighted to improve timescale robustness.

The purpose of the weighting strategy is to reflect the contribution of each onboard clock to long-term ensemble timekeeping, rather than to evaluate only short-term noise. For space-based timescale maintenance, clocks with good predictability over the update interval are more valuable than clocks that only appear quiet over short averaging times. Therefore, the weighting formulation in Equation (16) is designed to favor clocks with stable and predictable behavior, which is particularly suitable for onboard hydrogen masers with relatively smooth long-term evolution.

In addition, the timekeeping clocks used in this study are selected according to their in-orbit performance, including stability, data continuity, and long-term predictability. The goal is to construct a reliable and representative onboard ensemble for timescale generation, rather than to include all available clocks without screening.

The clock states and weights for the current interval are updated iteratively using the prediction and weighting models. When the difference between the ensemble clock products from two consecutive iterations falls below a preset threshold (e.g., 0.1 ns), TA(space) for this interval is produced. With high-precision inter-satellite synchronization, the method exploits the long-term stability of onboard hydrogen masers. It yields a continuous and predictable space-based timescale.

### 2.4. Space-Based Timescale Steering and Updating

Over long timescales, onboard atomic clocks are affected not only by phase and frequency white noise but also by colored noise such as frequency random walk. This causes the space-based timescale, when running autonomously, to accumulate an increasing offset relative to BDT. When satellite–ground links are available, we use the traceability clock offset from satellite–ground time transfer to update the predicted clock offset of the space-based timescale. This preserves the continuity of autonomous extrapolation. It also periodically corrects long-term drift and keeps the space-based timescale synchronized with BDT.

From a system-level perspective, the steering update is introduced to balance autonomous onboard timekeeping and long-term traceability to the ground reference. The space-based timescale is allowed to evolve autonomously between updates so that its short-term continuity and intrinsic stability are preserved, while periodic synchronization to BDT is used to suppress the gradual accumulation of long-term drift. Therefore, the steering process should be understood as an intermittent correction mechanism for cooperative timekeeping, rather than as a replacement for autonomous space-based timescale generation.

Let the ground time be TA(BDT). The satellite clock offsets from satellite–ground time transfer can be written as:

(17) $$X_{ground}=\left[\begin{array}{c} {clk}_{i}-TA(BDT)\\ \vdots \\ {clk}_{j}-TA(BDT) \end{array}\right]$$

The satellite clock offsets with respect to TA(space) are denoted by $X_{space}$. The offset between the space-based and ground timescales is:

(18) $${clk}_{diff}=X_{ground}-X_{space}=TA(BDT)-TA(space)$$

${clk}_{diff}$ characterizes the net offset of TA(space) relative to TA(BDT). It serves as a basic quantity for subsequent steering updates and consistency analysis.

To generate clock-product parameters and perform extrapolation, the long-arc clock offset of a BDS-3 hydrogen maser is modeled by a quadratic polynomial:

(19) $$y=a_{0}+a_{1}\cdot \left(t-t_{0}\right)+a_{2}\cdot {\left(t-t_{0}\right)}^{2}$$

Here, $a_{0}$, $a_{1}$, and $a_{2}$ represent phase, frequency, and frequency drift, respectively, and $t_{0}$ is the reference epoch. The clock offset series can be written in matrix form as:

(20) y=Ax+ε

Here,

(21) $$y=\left[\begin{array}{c} \begin{array}{c} y_{1}\\ y_{2}\\ \vdots \end{array}\\ y_{n} \end{array}\right],A=\left[\begin{array}{c} \begin{array}{c} 1\\ 1\\ \vdots \end{array}\\ 1 \end{array}\;\;\;\begin{array}{c} \begin{array}{c} {\left(t-t_{0}\right)}_{1}\\ {\left(t-t_{0}\right)}_{2}\\ \vdots \end{array}\\ {\left(t-t_{0}\right)}_{n} \end{array}\;\;\;\begin{array}{c} \begin{array}{c} {{\left(t-t_{0}\right)}_{1}}^{2}\\ {{\left(t-t_{0}\right)}_{2}}^{2}\\ \vdots \end{array}\\ {{\left(t-t_{0}\right)}_{n}}^{2} \end{array}\right],x=\left[\begin{array}{c} a_{0}\\ a_{1}\\ a_{2} \end{array}\right]$$

ε is the observation noise with covariance $Cov(\varepsilon )=\sigma_{0}^{2}P^{-1}$, where P is the weight matrix. For white noise, P=I. The least squares estimate is:

(22) $$\hat{a}={\left(A^{T}PA\right)}^{-1}A^{T}Py$$

At any prediction epoch t, let:

(23) $$\tau =t-t_{0},g(t)=\left[\begin{array}{ccc} 1 & \tau & \tau^{2} \end{array}\right]$$

The predicted clock offset is:

(24) $$\hat{y}(t)=g(t)\hat{a}$$

After $\hat{y}(t)$ is extrapolated for TA(space), new traceability data from satellite–ground time transfer may become available. We then update the prediction using $X_{ground}$. The updated value is used as the initial condition for the next extrapolation stage. The fit-arc length used for updating is configurable. We analyze how different arc length settings affect steering performance and clock predictability.

## 3. Performance Evaluation of BDS-3 Onboard Atomic Clocks

GNSS system time is realized by precise ground timing facilities and kept traceable to UTC. We examine whether, with improved onboard hydrogen masers and ISL time transfer accuracy, a constellation can establish and maintain a space-based timescale constrained by the ground. Using in-orbit BDS-3 inter-satellite data, this chapter quantifies how ISL network adjustment suppresses random noise in clock offset measurements and investigates the impact of high-precision relativistic corrections. We then evaluate the frequency stability and long-term predictability of the onboard hydrogen masers, providing a basis for timekeeping-ensemble selection, weight assignment, and steering strategies.

### 3.1. Random-Noise Assessment of Satellite Clock Offset Measurements After ISL Network Adjustment

BDS-3 Ka-band ISL provides highly redundant and precise two-way time transfer observations. However, each observation still contains measurement noise and unmodeled errors. To quantify how network adjustment suppresses random noise, we first examine the ISL measurement status. We then compare the clock offset noise levels before and after adjustment. The results are shown below.

**Figure 1:**
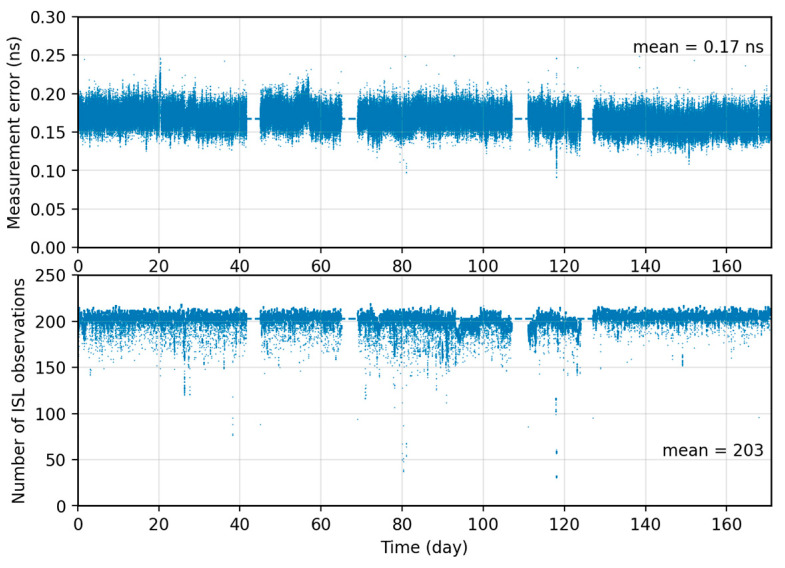
Time series of the ISL measurement error and the number of available ISL observations over the analysis period; the mean values are annotated.

Figure 1 shows the time-series characteristics of ISL observation quality over the analyzed arc. The upper panel shows the measurement error distribution. The mean is ~0.17 ns and remains stable over the arc. This indicates sub-nanosecond random noise with good temporal consistency. It is lower than the 0.21 ns fitting residual of ground-network clock products. The lower panel shows the number of available ISL observations per epoch. The mean is ~203 and stays at a high level. With a highly redundant network constraint, observation noise is effectively averaged down, yielding higher-precision clock offset series. Figure 1 indicates that the BDS-3 ISL observations provide not only sub-nanosecond measurement precision, but also a highly redundant inter-satellite observation network over the analyzed arc. This combination is important for constellation-level timekeeping because it provides a stable observational basis for suppressing single-link noise and improving the consistency of inter-satellite clock synchronization.

**Figure 2:**
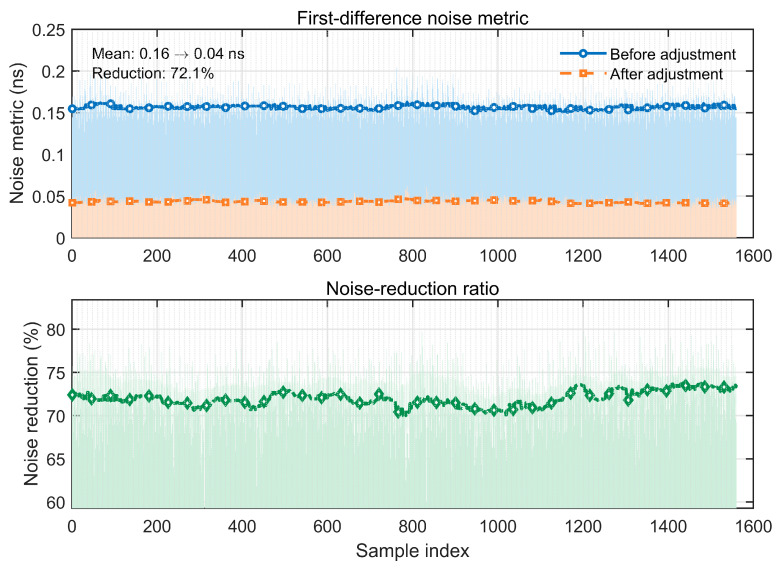
First-difference noise metric of the satellite clock offsets before and after ISL network adjustment, and the corresponding noise-reduction ratio at each epoch.

Because the RMS of raw clock offset residuals is sensitive to colored noise (e.g., random walk), we use a first-difference noise metric to quantify the reduction in inter-epoch high-frequency noise. As shown by the sample statistics in Figure 2, the mean first-difference noise decreases from 0.16 ns to 0.04 ns after network adjustment. This corresponds to an overall reduction of ~72.1%. The reduction is concentrated across samples and varies smoothly over the arc, indicating stable and consistent performance. Therefore, single-epoch network adjustment supported by highly redundant ISL observations can substantially reduce random noise. This improvement is important not only from a statistical point of view, but also because it directly enhances the quality of the relative clock-offset series used in the subsequent frequency–stability assessment, ensemble–timescale generation, and clock prediction. In other words, reducing the high-frequency noise floor at this stage helps establish a cleaner timing reference for the entire space-based timekeeping chain and makes the long-term behavior of the onboard clocks more observable and modelable.

### 3.2. Impact of Relativistic Correction on Long-Term Clock Offset Prediction

At sub-nanosecond time transfer accuracy, clock offset series are affected not only by measurement noise but also by residuals from the standard (ICD) relativistic correction. These residuals introduce periodic signatures that become increasingly visible. Over day-long timescales, they accumulate to sub-nanosecond errors. This degrades statistical stationarity and modelability, and it increases extrapolation error in long-term prediction. To quantify this effect, we use the post-adjustment clock offsets from Section 3.1 and compare the fitting and prediction results before and after applying the high-precision relativistic correction.

**Figure 3:**
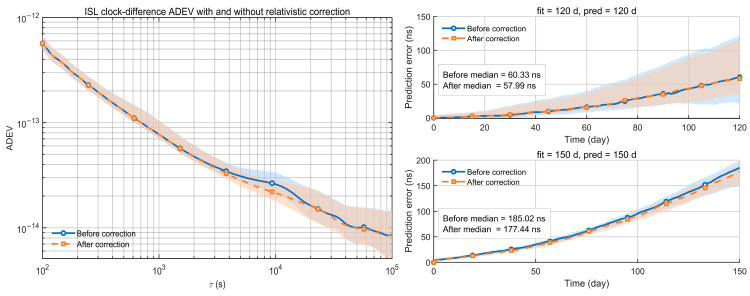
Allan deviation (ADEV) of ISL-based inter-satellite clock differences with and without the high-precision relativistic correction (**left**), and the corresponding long-term prediction errors for 120-day and 150-day fit–predict cases (**right**).

The left panel of Figure 3 shows the Allan deviation (ADEV) of inter-satellite clock offsets with and without relativistic correction. The blue curve is the median before correction, and the red curve is the median after correction. The shaded bands denote the interquartile range (IQR). The high-precision correction removes the day-scale periodic signature and stabilizes the short-term frequency behavior. Around $\tau \approx 10^{4}$ s, space-based ensemble atomic timescale $2.60\times 10^{-14}$ to $2.14\times 10^{-14}$, an improvement of 17.77%.

The right panel of Figure 3 shows rolling prediction errors of the clock offset series. Blue and red curves are the medians before and after correction, respectively. The shaded areas indicate the IQR. For long-horizon prediction, the correction accumulatively reduces the error by several to tens of nanoseconds. The 120-day error decreases from 60.33 ns to 57.99 ns, and the 150-day error decreases from 185.02 ns to 177.44 ns.

Therefore, once the ISL network adjustment has largely suppressed random noise, residual relativistic model errors become a non-negligible contributor. Applying the high-precision correction improves day-scale stability and further reduces long-term prediction error. From a system perspective, this means that, at sub-nanosecond inter-satellite time transfer accuracy, accurate physical modeling becomes an essential part of autonomous timekeeping performance. Otherwise, uncorrected periodic relativistic residuals would degrade the modelability of the clock-offset series and accumulate into larger extrapolation errors over long prediction horizons.

### 3.3. Frequency Stability Evaluation of Onboard Hydrogen Masers

Using clock offset series after ISL network adjustment and the high-precision relativistic correction, we evaluate the frequency stability of BDS-3 onboard hydrogen masers. Hydrogen masers exhibit measurable frequency drift. The Hadamard deviation (HDEV) is insensitive to drift and provides a more robust measure of mid- to long-term stability. It is therefore suitable for day-scale and longer assessments in this study. For clarity, all satellite identifiers in this paper are expressed in standard PRN notation. We select C26 as the reference clock because of its stable operation and good data completeness. We form a clock offset series for each satellite relative to C26. HDEV is then computed over a 180-day arc, as shown below.

**Figure 4:**
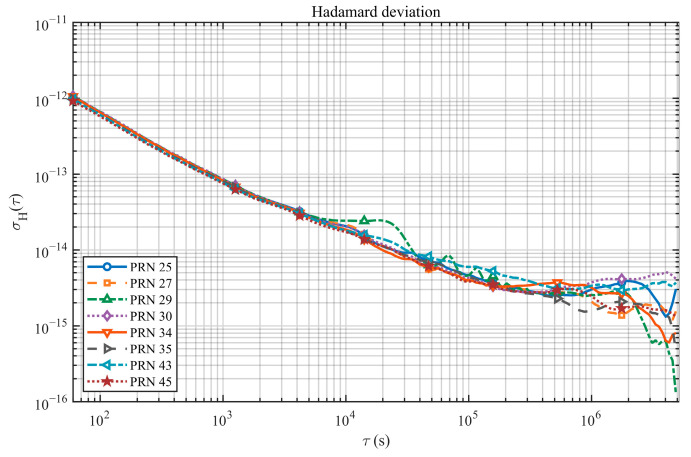
HDEV of BDS-3 onboard hydrogen masers (relative to the reference clock C26).

As shown in Figure 4, all curves exhibit a consistent power-law decay at short to mid averaging times. Over $\tau =10^{2}-10^{4}$ s, HDEV decreases from $∼10^{-12}$ to $∼10^{-14}$. Around $\tau \approx 10^{5}$ s, it further reaches the $∼10^{-15}$ level. Differences among satellites are small overall, indicating good consistency in stability across the selected hydrogen masers.

Table 1 lists the HDEV values at representative averaging times. Note that our stability assessment is based on inter-satellite clock-difference series. For BDS-3 hydrogen masers with comparable performance, the equivalent single-clock HDEV can be approximated from the two-clock difference (typically scaled by $1/\sqrt{2}$). Therefore, the day-level stability of a single hydrogen maser currently reaches the $∼3\times 10^{-15}$ level.

### 3.4. Long-Term Prediction Accuracy of Onboard Hydrogen Masers

Space-based autonomous timekeeping requires not only good short-term stability, but also long-term predictability. This is essential for maintaining a timescale without external constraints. In this section, we statistically evaluate the prediction accuracy of the clock offset series. The results are given below.

Short-term prediction results are summarized in Table 2. Here, RMS is reported as the mean ± standard deviation of the RMS values computed from sliding windows. END is the mean ± standard deviation of the end-point prediction error. The 2 h prediction uses linear extrapolation with a 1-day fit arc. The 1-day prediction uses a quadratic model with the same 1-day fit arc. Results show that the 2 h prediction has an RMS of about 0.1 ns. Differences among satellites are negligible, and the mean END stays close to zero. For the 1-day prediction, the RMS is about 0.8 ns, and the mean END is still near zero. This level is comparable to short-arc clock prediction under nanosecond-level satellite–ground time transfer noise. It indicates that, for sub-day horizons, prediction errors are mainly driven by frequency white noise and measurement noise.

**Figure 5:**
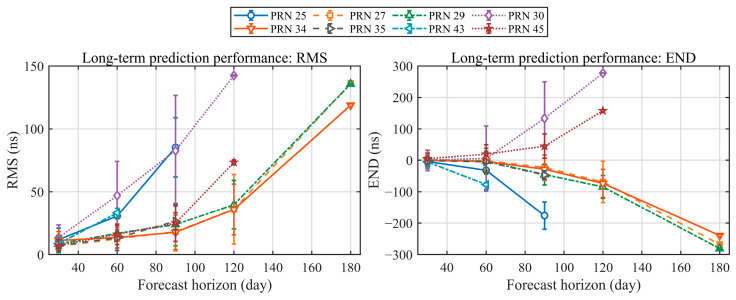
Long-term prediction performance of onboard hydrogen masers, evaluated by the RMS (**left**) and end-point error (END; **right**) of the clock offset predictions at different prediction horizons.

Long-term prediction performance is shown in Figure 5. It reports the mean ± standard deviation of RMS and END over prediction horizons from 30 to 180 days. Long-term prediction uses a quadratic model. The prediction horizon equals the fit-arc length. Because the available data arcs are limited, the effective sample size is small for the 120-day and 180-day cases. Overall, RMS increases nonlinearly with the prediction horizon. It reaches the order of tens of nanoseconds at 30 days. At 120 days, it rises to several tens of nanoseconds for most satellites and exceeds 100 ns for some. Meanwhile, as the horizon increases, the mean END for some satellites drifts away from zero. It shows a persistent negative or positive bias, and the magnitude grows with the horizon. These results suggest that, over long timescales, errors in drift-rate estimation, time-varying drift, and unmodeled higher-order terms accumulate during extrapolation. They lead to larger prediction errors and stronger inter-satellite differences.

## 4. Experiments and Discussion

To evaluate the performance of the proposed timekeeping framework under different operational conditions, we design two representative timescale scenarios. The first is a free-running space-based timescale, denoted TA(SPACE)-free, which represents a pure onboard autonomous timekeeping mode supported only by onboard hydrogen masers and inter-satellite links. This scenario is intended to assess the intrinsic capability of the constellation to maintain a self-consistent timescale without ground support. The second is a ground-steered space-based timescale, denoted TA(SPACE)-steer, which represents a space–ground cooperative timekeeping mode in which the space-based timescale is periodically synchronized to BDT. This scenario is intended to assess how intermittent ground support can suppress long-term drift while preserving the short-term stability advantage of the space-based timescale. In addition, steering intervals of 10, 20, and 30 days are compared to analyze the trade-off between ground synchronization strength and autonomous timescale continuity. We also present satellite clock prediction results that refer to the space-based timescale, thereby providing a comprehensive validation of both autonomous and cooperative timing performance.

### 4.1. Space-Based Timescale Based on Onboard Hydrogen Masers

In this subsection, we analyze the free-running space-based timescale, denoted TA(SPACE)-free, to assess the capability of pure onboard autonomous timekeeping. We select the onboard hydrogen masers on satellites 26, 27, 29, 44, and 52 to form the timekeeping ensemble. All satellites are initially synchronized to BDT through satellite–ground traceability, after which the ensemble clocks are propagated using a quadratic model and combined according to the algorithm in Section 2.3. No further ground steering is applied during the evaluation interval. Therefore, this experiment represents a purely onboard timekeeping scenario and is used to quantify the stability and long-term drift behavior of the space-based timescale relative to BDT. The results are presented below.

**Figure 6:**
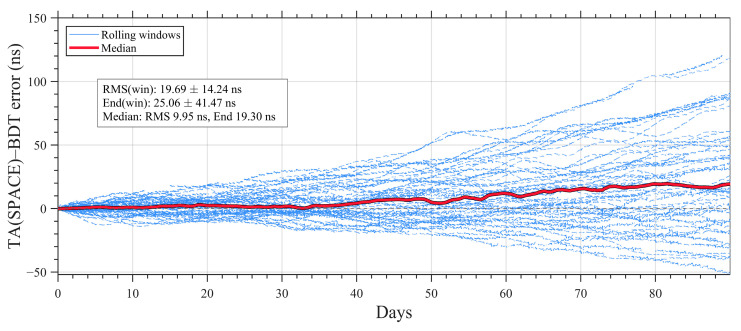
The time difference between TA(SPACE) and BDT.

Figure 6 shows the rolling statistics of the time difference between TA(SPACE) and BDT. The blue curves represent the time difference series from individual rolling windows, and the red curve denotes the median trajectory. In the free-running mode, the TA(SPACE)–BDT difference exhibits a systematic drift that accumulates over time. Over the 90-day arc, the RMS of the rolling-window time difference is 19.69 ± 14.24 ns, and the end-point bias (End) is 25.06 ± 41.17 ns. Using the median trajectory as an overall representative, the RMS and end-point bias are approximately 9.95 ns and 19.30 ns, respectively.

**Figure 7:**
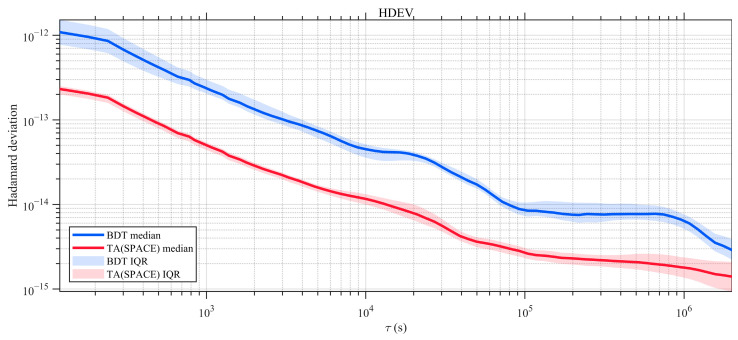
HDEV of satellite clocks referenced to TA(SPACE) and to BDT.

To further examine the frequency stability of the space-based timescale in the free-running mode, Figure 7 presents the HDEV of TA(SPACE) and BDT. At short averaging times, TA(SPACE) is clearly more stable than a single onboard atomic clock. This indicates that the ensemble effectively suppresses phase and frequency white noise. As the averaging time increases, the TA(SPACE) stability curve gradually approaches the long-term stability level of the onboard hydrogen masers. This suggests that, although ensemble averaging reduces random noise, frequency random walk and biases in drift-rate estimation—together with their slow temporal variations—become dominant over long timescales. As a result, the long-term performance is ultimately limited by the intrinsic behavior of individual clocks.

Taken together, the time difference and stability results show that the space-based ensemble atomic timescale has good short- to mid-term continuity and stability without external constraints. However, its long-term offset relative to the ground time reference inevitably accumulates over time. Therefore, to maintain long-term consistency between the space-based timescale and the ground system time, periodic external constraints or steering are required. This is also necessary when satellite–ground links are limited and system–time drift must be controlled. Based on this, the next section analyzes space–ground cooperative autonomous timekeeping enabled by periodically updating TA(SPACE) under intermittent satellite–ground link availability.

### 4.2. Space–Ground Cooperative Autonomous Timekeeping

As verified in Section 4.1, the free-running space-based timescale can maintain good short- and mid-term stability, but its offset relative to BDT gradually accumulates over long timescales. To investigate how intermittent ground support can mitigate this drift, we analyze a periodically steered space-based timescale, denoted TA(SPACE)-steer. This mode represents a space–ground cooperative timekeeping scenario in which the constellation preserves its onboard timing autonomy while being periodically constrained by the ground reference. We consider steering intervals of 10, 20, and 30 days to examine how the update frequency affects the trade-off between long-term synchronization to BDT and the preservation of the intrinsic stability of the space-based timescale. Smaller intervals correspond to stronger ground constraints, whereas larger intervals correspond to greater onboard autonomy. This comparison helps clarify the applicable operating regime of cooperative timekeeping in future integrated PNT systems. At each update, the available external reference is used to correct the drift of the space-based timescale. The updated timescale is then used as the initial condition for the next free-extrapolation stage. The results are shown below.

**Figure 8:**
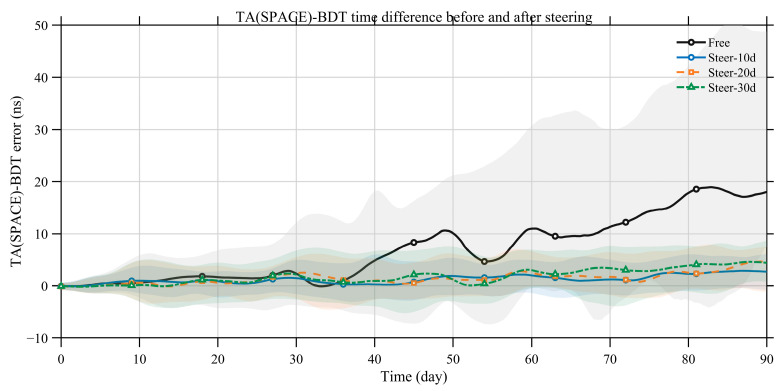
TA(SPACE)–BDT difference before steering (free-running) and after BDT steering with update intervals of 10, 20, and 30 days (median trajectories with IQR bands).

As shown in Figure 8, steering TA(SPACE) markedly reduces the growth rate of the TA(SPACE)–BDT difference compared with the free-running case, and the fluctuation range is much smaller. Table 3 further indicates that the steered TA(SPACE) can be maintained within 10 ns relative to BDT. Moreover, more frequent steering leads to a smaller TA(SPACE)–BDT offset.

**Figure 9:**
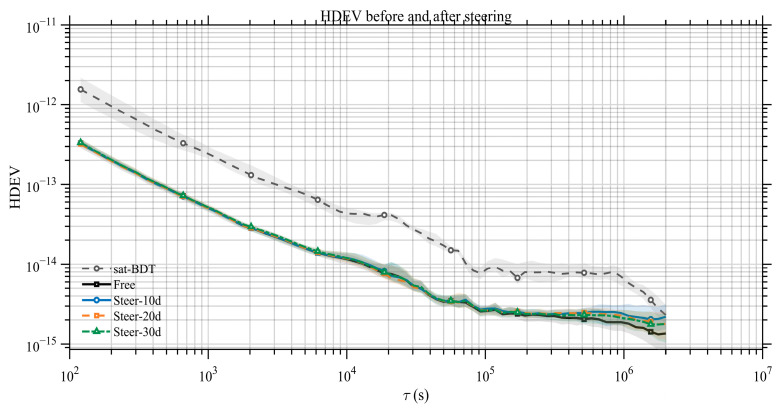
HDEV of satellite clocks referenced to TA(SPACE) before steering (free-running) and after BDT steering with update intervals of 10, 20, and 30 days (median curves with IQR bands).

Figure 9 and Table 3 show that, over short timescales, the steered space-based timescale behaves similarly to the free-running case. Over long timescales, as the steering interval decreases, the long-term stability progressively shifts from the free-running performance toward the BDT-referenced satellite-clock performance. This indicates that more frequent steering drives the long-term behavior closer to the reference timescale.

In summary, for a free-running space-based timescale, intermittent satellite–ground link availability enables periodic steering. This can effectively suppress drift relative to the ground time reference and maintain space–ground time synchronization.

### 4.3. Clock Offset Prediction Performance of the Space-Based Timescale

In applications of onboard time references, short-horizon clock offset prediction is critical. It affects the maintenance of inter-satellite time synchronization and the performance of user positioning and timing. To assess hour-scale predictability, we use a 2 h prediction window. We compare clock offset prediction results referenced to TA(SPACE) and to BDT.

**Figure 10:**
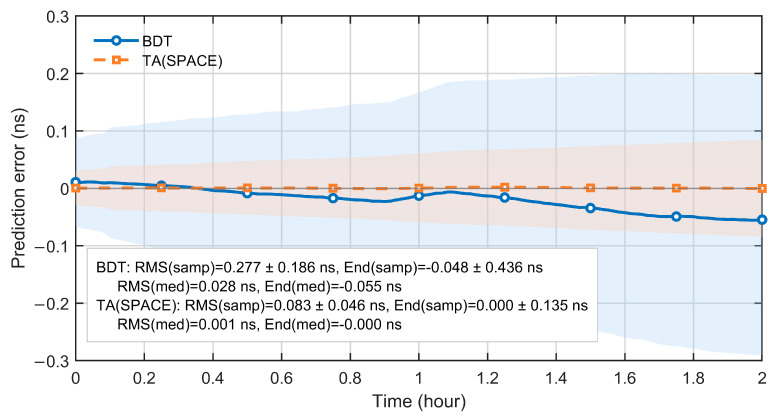
Statistics of 2 h satellite clock offset prediction errors referenced to TA(SPACE) and to BDT (median curves with IQR bands).

Figure 10 summarizes the clock offset prediction errors over a 2 h window when using TA(SPACE) and BDT as the reference timescales. The median and interquartile range (IQR) are reported to describe the central tendency and dispersion. Over the full 2 h horizon, the TA(SPACE)-referenced errors remain close to zero. The median stays near 0 ns, and the IQR is smaller than that under BDT. In terms of summary metrics, both the sample RMS and the end-point error under TA(SPACE) are significantly lower than those under BDT. This indicates that, at short timescales, a space-based timescale more effectively suppresses the impact of random noise and slowly varying components on clock prediction. This advantage mainly comes from the high-precision relative clock offset information provided by inter-satellite time transfer, which gives TA(SPACE) stronger internal consistency over short intervals.

**Figure 11:**
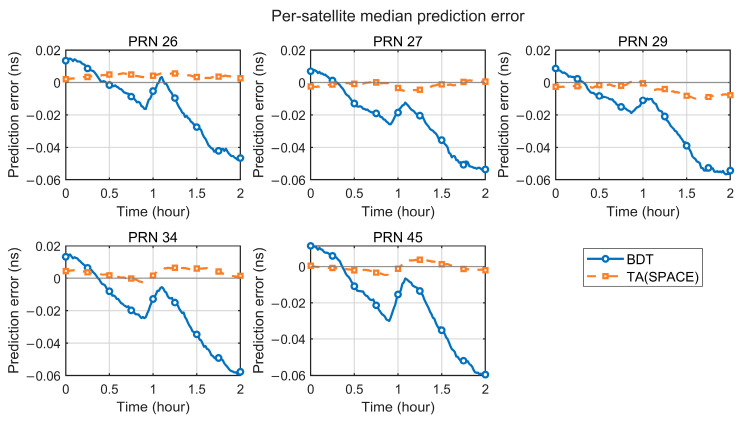
Two-hour satellite clock offset prediction errors for individual satellites, referenced to TA(SPACE) and to BDT (per-satellite median curves).

To further examine short-horizon prediction consistency across satellites, Figure 11 compares the per-satellite median errors over the 2 h window. With TA(SPACE) as the reference, the prediction errors evolve consistently across satellites. The curves fluctuate slightly around zero. No pronounced systematic drift is observed. In contrast, BDT-referenced predictions exhibit a coherent one-sided drift across satellites. The error magnitude increases with the prediction horizon.

These results show that, over a 2 h horizon, TA(SPACE) provides better prediction performance than the ground reference time. Together with the results in Section 4.1 and Section 4.2, TA(SPACE) demonstrates strong performance at short, mid, and long timescales. It supports high-quality autonomous timekeeping.

## 5. Conclusions

This study develops and validates a methodological framework for establishing, maintaining, and predicting a space-based time reference, leveraging the onboard hydrogen masers of BeiDou-3 (BDS-3) and its inter-satellite and satellite–ground time transfer capabilities. Using long-term in-orbit clock offset measurements and ISL data, we systematically evaluate the performance of onboard hydrogen masers, the accuracy of inter-satellite time transfer, and the space-based timescale performance under different operating modes. The main conclusions are summarized as follows.

(1) The BDS-3 onboard hydrogen masers exhibit high stability and good long-term predictability. The ISL observation noise is about 0.17 ns; using constellation-wide network adjustment with ~203 ISL measurements reduces it to ~0.04 ns (a 72.1% reduction). High-accuracy relativistic correction improves the short-term frequency stability of the inter-satellite clock-difference series: at ~10,000 s, the stability improves from ~2.60×10^−14^ to 2.14×10^−14^ (Allan), i.e., by ~17.77%, and the day-level stability reaches 3×10^−15^ (Hadamard). Based on these results, the intra-day clock offset prediction error of a single satellite can be stably controlled at the nanosecond level, while the long-term prediction error remains at the sub-100 ns level for timescales of 30 days and longer. These results indicate that both noise suppression and high-fidelity physical modeling are indispensable for building a practical space-based timescale, because they improve not only the apparent precision of the clock products, but also the stability, predictability, and operational usability of the autonomous timing reference.
(2) By introducing an ALGOS-like predict–weight–iterate scheme and using high-precision inter-satellite clock differences, we construct the space-based timescale TA(SPACE). Without external constraints, TA(SPACE) can be maintained continuously and self-consistently. Experiments show that, over a 90-day autonomous run, the time offset of TA(SPACE) relative to ground-based BDT remains within 80 ns, with an RMS of 19.69 ± 14.24 ns and an end-point bias (End) of 25.06 ± 41.47 ns. Its stability is better than that of a single clock, demonstrating that multi-clock averaging effectively suppresses random noise.
(3) When satellite–ground links are available, periodically steering TA(SPACE) using the ground time reference effectively suppresses the accumulation of long-term extrapolation errors. Space–ground cooperative experiments with steering intervals of 10, 20, and 30 days show that the TA(SPACE)–BDT offset can be kept within 10 ns after steering. As the steering interval decreases, the long-term stability gradually approaches that of the BDT-referenced satellite clock, reflecting the increasing influence of the reference timescale.
(4) The space-based time reference shows excellent predictability at the hourly scale. In two-hour prediction tests, clock offset predictions referenced to TA(SPACE) remain close to 0 ns with much smaller dispersion than those referenced to ground-based BDT. The results are highly consistent across satellites, indicating that the ISL-enabled space-based reference effectively suppresses random noise at short timescales.

In summary, the in-orbit performance of BDS-3 onboard hydrogen masers and inter-satellite time transfer links is robust, providing solid support for the construction, maintenance, and prediction of a space-based time reference. With the proposed space-based timescale algorithm and steering strategy, a smooth transition from space–ground cooperative timekeeping to space-based autonomous timekeeping can be achieved under varying satellite–ground link availability. This improves the resilience of GNSS time services and enhances constellation autonomy, while maintaining service continuity and accuracy.

Future work will further extend the proposed framework toward more realistic and resilient system-level operation. First, the present study uses a small ensemble of onboard hydrogen masers for timekeeping. In the future, we will expand the ensemble size and investigate constellation-wide participation of onboard clocks, so as to improve the robustness and redundancy of space-based timekeeping. Second, we will study adaptive weight adjustment strategies for degraded service scenarios, such as failure of a small number of onboard clocks or interruption of a limited number of inter-satellite links, in order to maintain stable timescale performance under partial system damage. Third, we will further investigate effective identification and isolation methods for abnormal information, including spoofed measurements and failed onboard clocks, to ensure the smooth and reliable operation of the timing system in the presence of disturbances. By considering a wider range of realistic scenarios, the proposed method can be further developed into a resilient timekeeping architecture capable of maintaining continuous service under different operational conditions.

## Figures and Tables

**Table 1:** HDEV of BDS-3 onboard hydrogen masers.

Satellite	HDEV_10,000 s/×10^−14^	HDEV_1 d/×10^−15^	HDEV_30 d/×10^−15^
25	2.06	4.98	3.50
27	2.12	4.24	1.81
29	2.43	4.86	1.19
30	2.02	4.54	4.22
34	1.85	4.30	1.63
35	1.77	4.78	1.87
43	1.80	6.17	3.13
45	1.70	4.00	1.79

**Table 2:** Short-term prediction accuracy evaluation.

PRN	RMS_2 h/ns	RMS_1 d/ns	END_2 h/ns	END_1 d/ns
25	0.11 ± 0.06	0.85 ± 0.64	0.00 ± 0.16	0.24 ± 2.01
27	0.11 ± 0.06	0.70 ± 0.47	0.00 ± 0.16	−0.05 ± 1.55
29	0.14 ± 0.07	0.89 ± 0.57	0.00 ± 0.22	−0.06 ± 1.87
30	0.11 ± 0.05	0.73 ± 0.48	0.01 ± 0.16	0.03 ± 1.62
34	0.11 ± 0.05	0.72 ± 0.50	0.00 ± 0.15	−0.09 ± 1.63
35	0.10 ± 0.05	0.80 ± 0.60	0.00 ± 0.15	−0.07 ± 1.90
43	0.11 ± 0.06	1.05 ± 0.71	0.00 ± 0.17	0.01 ± 2.42
45	0.10 ± 0.05	0.72 ± 0.52	0.00 ± 0.14	0.16 ± 1.62

**Table 3:** Performance comparison of TA(SPACE) before and after BDT steering.

Scenario	RMS (Win)/ns	END (Win)/ns	RMS (Med)	END (Med)	HDEV (τ = 1 d) (×10^−15^)	HDEV (τ = 10 d) (×10^−15^)
Free	21.22 ± 16.17	28.57 ± 45.42	10.57	21.15	2.92	1.46
Steer-10 d	4.60 ± 1.86	2.62 ± 5.05	1.80	3.01	3.03	2.52
Steer-20 d	5.48 ± 2.51	3.43 ± 6.50	2.18	4.36	3.01	2.31
Steer-30 d	6.38 ± 2.90	3.77 ± 7.43	2.65	3.37	3.02	2.22

## Data Availability

The original contributions presented in this study are included in the article. Further inquiries can be directed to the corresponding author.
